# Peptide derived from progranulin of the carcinogenic liver fluke, Opisthorchis viverrini stimulates cell hyperproliferation and proinflammatory cytokine production

**DOI:** 10.21203/rs.3.rs-2586058/v1

**Published:** 2023-03-14

**Authors:** Thanapat Hembasat, Sujittra Chaiyadet, Wannaporn Ittiprasert, Michael J Smout, Neil D Young, Alex Loukas, Paul J Brindley, THEWARACH LAHA

**Affiliations:** Khon Kaen University Faculty of Medicine; Khon Kaen University Faculty of Medicine; GWU: George Washington University; James Cook University - Cairns Campus; The University of Melbourne; James Cook University - Cairns Campus; GWU: George Washington University; Khon Kaen University Faculty of Medicine

**Keywords:** Opisthorchis viverrini, liver fluke, progranulin, cholangiocyte, cytokines

## Abstract

**Purpose:**

Progranulin (PGRN) is a secreted glycoprotein growth factor with roles in wound healing, inflammation, angiogenesis and malignancy. An orthologue of the gene encoding human PGRN was identified in the carcinogenic liver fluke *Opisthorchis viverrini*.

**Methods:**

Sequence structure, general characteristics and possible function of *O. viverrini* PGRN was analyzed using bioinformatics. Expression profiles were investigated with quantitative RT-PCR, western blot and immunolocalization. A specific peptide of *Ov*-PGRN was used to investigate a role for this molecule in pathogenesis.

**Results:**

The structure of the gene coding for *O. viverrini* PGRN was 36,463 bp in length, and comprised of 13 exons, 12 introns, and a promoter sequence. The *Ov-pgrn* mRNA is 2,768 bp in length and encodes an 846 amino acids with a predicted molecular mass of 91.61 kDa. *Ov*-PGRN exhibited one half and seven complete granulin domains. Phylogenetic analysis revealed that *Ov*-PGRN formed its closest relationship with PGRN of liver flukes in the Opisthorchiidae. Transcripts of *Ov-pgrn* were detected in several developmental stages, with highest expression in the metacercaria, indicating that *Ov*-PGRN may participate as a growth factor in the early development of *O. viverrini*. Western blot analysis revealed the presence of detected *Ov*-PGRN in both soluble somatic or excretory/secretory products, and immunolocalization indicated high levels of expression in the tegument and parenchyma of the adult fluke. Co-culture of a human cholangiocyte cell line and a peptide fragment of *Ov*-PGRN stimulated proliferation of cholangiocytes and upregulation of expression of the cytokines IL6 and IL8.

**Conclusion:**

Ov-PGRN is expressed throughout the life cycle of liver fluke, and likely plays a key role in development and growth.

## Introduction

Infection with the food-borne liver fluke, *Opisthorchis viverrini* has been classified as a group 1 biological carcinogen by the International Agency for Research on Cancer [1, 2]. In regions where liver fluke infection is endemic, opisthorchiasis is the principal risk factor for cholangiocarcinoma (CCA) [3]. Focusing on the contribution of the excretory and secretory products (ES) of *O. viverrini* to carcinogenesis, we targeted the *O. viverrini* granulin-like growth factor, *Ov*-GRN-1, a prominent component of the ES complement that induces phenotypic hallmarks of cancer. *Ov*-GRN-1 and other ES components including extracellular vesicles enter cholangiocytes, the epithelial cells that line the biliary tract, and drive cellular signaling that promotes carcinogenesis, including cellular proliferation and migration, angiogenesis and wound healing [4–6]. We have confirmed the role of *Ov*-GRN-1 in driving proliferation of bile duct epithelial cells (cholangiocytes) by genetic manipulation of its expression in the liver fluke both by RNA interference and CRISPR gene knockout. Moreover, we have shown that infection of hamsters with the gene-edited, infectious stage of the live fluke was feasible and that proliferation of biliary epithelia is markedly suppressed during infection with the *ΔOv-grn-1* (*Ov-grn-1* knockout) flukes [4, 6–9].

The genome of *O. viverrini*encodes three granulin-like genes including progranulin (*Ov*-PGRN) and two opisthorchiid-specific granulins (*Ov*-GRN-1 and *Ov*-GRN-2) that were identified in *in silico* generated ES products of *O. viverrini* [10]. Mammalian PGRN has been studied in depth; it is a secreted glycoprotein comprised of multiple granulin domains. Each granulin domain shows 12 conserved cysteine granulin/epithelin modules [11, 12]. Cleavage of the signal peptide releases the mature granulin, which can be further cleaved into a slew of active, 6 kDa peptides [13]. The granulin/epithelin module (GEM) itself (contained within each unit) and intact PGRN protein can both regulate cellular proliferation [14, 15]. The majority of the granulin family members across the web of life are in a multi-domain form mostly commonly as multiple granulin domains, similar to PGRN.

*Opisthorchis* and related species are unusual with single domain granulin genes, the most studied form being *Ov*-GRN-1 which has been shown to promote cell proliferation and wound healing [8, 16–18]. Human PGRN plays a notable role in stimulating cellular proliferation and inflammation, which in turn promotes tumor metastasis [13, 19]. Human PGRN is essential for a range of organs, and mutations lead to dementia-like disease in the brain [20].

Notwithstanding that *Ov*-GRN-1 has been studied extensively, *Ov-pgrn* from any of the liver flukes has yet to be investigated. Here, we describe the characteristics of the *Ov*-PGRN protein and investigate the properties of a synthetic peptide of *Ov*-PGRN-1 that lacks identity to the orthologous human protein and induced proliferation of the H69 cholangiocyte cell line, a model for normal biliary tract epithelium, and induced expression of cholangiocyte proinflammatory cytokines.

## Materials And Methods

### Ethics statement

Hamsters were reared at the animal facility, Faculty of Medicine, Khon Kaen University. Study design protocols and standard operating procedures were approved by the Animal Ethics Committee of Khon Kaen University according to the Ethics of Animal Experimentation of the National Research Council of Thailand, approval number IACUC-KKU-92/63.

### Developmental stages of *O. viverrini*

*O. viverrini* metacercariae were isolated from infected cyprinid fishes from natural sources by pepsin digestion as described (Pinlaor et al., 2013). Newly excysted-juvenile flukes (NEJ) were prepared from the encysted metacercariae by incubation in 0.25% trypsin in 1’ PBS supplemented with 2’ 200U/ml penicillin, 200 mg/ml streptomycin (Gibco, Thermo Fisher Scientific, Waltham, MA) for five min at 37°C in 5% CO_2_ in air, after which NEJs were separated from the discarded cyst walls using mechanical passage through a 24G gauge needle. Flukes were recovered from the biliary tract of Syrian golden hamsters (*Mesocricetus auratus*) at 2 weeks and 6 weeks after infection with 50-100 metacercariae per hamster by intragastric intubation, to recover juvenile and adult stages of the helminth, respectively [21].

### Somatic adult extract (SAE) and excretory-secretory (ES) antigen preparation

A soluble lysate of adult worms or somatic adult worm extract (SAE) was prepared from fresh or frozen and homogenized adult worms as described [8]. Briefly, adult worms (~20 worms) recovered from hamsters (above) were washed in cold normal saline solution (NSS) containing 100 μg/ml penicillin-streptomycin. The worms were frozen in liquid nitrogen and homogenized with glasses tissue grinder in PBS containing 1X proteases inhibitor. Lysate was lysed with ultrasonic homogenizer with amplitude 20% at 20 pulses/min for 10 min and centrifuged 10,000×g for 30 min at 4°C. Supernatant was collected and concentration of protein determined by spectrophotometry at 280 nm. SAE was aliquoted and kept at −80°C until used.

For preparation of the excretory-secretory (ES) products of adult *O. viverrini*, the live adult worms (~200 worms) collected from experimental hamsters above were washed in sterile NSS containing antibiotics (100 μg/ml penicillin-streptomycin) and transferred into RPMI 1640 culture medium (Gibco, Grand Island, NY) supplemented with 1% glucose, antibiotics and proteinases inhibitors and maintained in culture medium (200 μl/fluke) at 37°C, under 5% CO_2_ in air. After incubation for 24 hr, the medium containing ES products was removed, clarified by centrifugation at 3,000×g at 4°C for 10 min, the supernatant was concentrated using Amicon 8050 ultrafiltration cell (Amicon, Miami, FL) equipped with a YM10 membrane (cutoff, 10,000 daltons), dialyzed against phosphate-buffered saline (PBS) pH 7.4, and sterilized by 0.22 mm filtration. The protein concentration was measured as above after which aliquots were stored at −80°C [22].

### Phylogenetic analysis

Progranulin protein sequences were retrieved from GenBank. Sequence homologies were analyzed using the BLAST search program [23]. Signal peptide and cleavage site was predicted with SignaIP - 5.0 (https://services.healthtech.dtu.dk/service.php?SignaIP-5.0). Promoter sequence and transcription site was predicted at https://www.fruitfly.org/seq_tools/promoter.html. Asparagines predicted to be N-glycosylation sites were analyzed at https://services.healthtech.dtu.dk/service.php?NetNGlyc-1.0. The protein sequences of PGRN from diverse taxa were retrieved and compared to PGRN of *O. viverrini* by ClustalW multiple sequences alignment analysis using BioEdit version 7.2.6 [24, 25]. The phylogenetic tree of PGRN protein sequences was analyzed and constructed with Maximum likelihood method with Jones-Taylor-Thornton (JTT) model with 1,000 bootstrap replication by using software MEGA version 11.0 [26]. Progranulin protein sequences in phylogenetic tree including THD19177.1 *Fasciola hepatica*, TPP59761.1 *Fasciola gigantica*, KAA0196540.1 *Fasciolopsis buski*, VDP74698.1 *Echinostoma caproni*, CDS24124.1 *Echinococcus granulosus*, CDI98749.1 *Echinococcus multilocularis*, VUZ39807.1 *Hymenolepis diminuta*, CAH8605806.1 *Schistosoma intercalatum*, CAH8636088.1 *Schistosoma haematobium*, CAH8621661.1 *Schistosoma bovis*, XP_018648777.1 *Schistosoma mansoni*, CAX73857.1 *Schistosoma japonicum*, CAH8659303.1 *Dicrocoelium dendriticum*, KAA3673437.1 *Paragonimus westermani*, KAF6780227.1 *Paragonimus kellicotti*, KAF5405781.1 *Paragonimus heterotremus*, TGZ71538.1 *Opisthorchis felineus*, KAG5441482.1 *Clonorchis sinensis*, XP_009174632.1 *Opisthorchis viverrini*, KRX87044.1 *Trichinella pseudospiralis*, XP_003371171.1 *Trichinella spiralis*, XP_024506721.1 *Strongyloides ratti*, KFIN88158.1 *Toxocara canis*, NP_492981.1 *Caenorhabditis elegans*, XP_035590678.1 *Oncorhynchus keta* (chum salmon), XP_028930903.1 *Ornithorhynchus anatinus* (platypus), AAI05335.1 *Bos Taurus* and NP_002078.1 *Homo sapiens*.

### A liver fluke specific peptide fragment of PGRN

The peptide LQSKKDISDAFIRMQC (amino acids position 627 – 641) designated “*Ov*-PGRN-L627C641” (Supporting information, Fig. S1) was selected because it appeared to be liver fluke PGRN-specific (does not share identity to human PGRN) and was predicted to be highly immunogenic in rabbits, and therefore suitable for raising a specific polyclonal antiserum. The peptide was synthesized and was used to generate the polyclonal anti-*O*. *viverrini* progranulin antibodies by immunizing New Zealand rabbits (Genscript, Piscataway, NJ).

### RNA extraction and quantitative real-time RT-PCR

Total RNA of flukes was extracted with TRIZOL reagents (ThermoFisher Scientific, Burlington, MA) following the manufacturer’s instructions, and contaminating genomic DNA was removed by treatment with DNase I (ThermoFisher Scientific, Rockfort, IL). The RNA concentration was measured by Nanodrop 2000 (ThermoFisher Scientific, Wilmington, DE). cDNA was converted from 500 ng of flukes total RNA by using a RevertAid first strand cDNA synthesis kit (Thermo Fisher Scientific, Waltham, MA) for qPCR templates. qPCR was performed with biological duplicate samples using a SYBR Green kit (Takara Bio USA, Inc., Mountain View, CA) in a thermal cycler (Light Cycler 480 II, Roche Diagnostics GmbH, Mannheim, Germany). Each qPCR reaction consists of 1 μL of cDNA, 10 μL SYBR Green Master Mix, 10 mM forward and reverse primers for specific *Ov-pgrn* gene (forward primer, *Ov*-PGRN-out-f: 5’-TGTCGGTTCGGGATCCATTG and reverse primer, *Ov*-PGRN-rev: 5’-ACTTACATTAACGAAAGGACAGC), and distilled water to a final volume of 20 μL. The thermal cycle was started with a single initiation cycle at 95°C for 10 minutes. The cycle was followed by 40 cycles quantification mode of PCR, each PCR cycle consists of denaturation at 95°C for 15 sec, annealing at 53°C, single acquisition mode for 30 sec, and extension at 72°C for 30 sec. After the final PCR step, the specificity of the real-time PCR reaction was confirmed using melting curve analysis. The evaluation of PGRN expression was performed by relative gene expression analysis using housekeeping genes. The endogenous actin gene (GenBank EL620339.1) was used as a reference control [21]. The control group was prepared the same qPCR reaction as described above except for the difference in a pair of primers for liver fluke actin gene instead (forward primer, *Ov*-actin-F1: 5’-AGCCAACCGAGAGAAGATGA, and reverse primer *Ov*-actin-R1: 5’-ACCTGACCATCAGGCAGTTC). The relative *Ov-pgrn* mRNA expression from different developmental stages of *O. viverrini*, the fold change (R) was calculated by, where ΔCt = Ct value of *Ov-pgrn* 𠄼 Ct of value *Ov*-actin [28].

### Western blot analysis

To detect PGRN in somatic adult worm extract and ES product of *O. viverrini*, 2 mg of SAE and 5 mg ES products were separated in 15 % SDS-PAGE and probed with anti-*Ov*-PGRN-L627C641 peptide. In brief, electrophoresed proteins were transblotted onto nitrocellulose membrane, after which the membrane was cut into strips and blocked for non-specific binding with 5 % skim milk in PBST for 1 hr. The membranes were first incubated overnight with purified polyclonal anti-*O*. *viverrini* progranulin antibodies generated by immunizing a New Zealand rabbit with the peptide *Ov*-PGRN-L627C641 (see above) at dilution 1:300 then incubated for 120 min in goat anti-rabbit IgG conjugated with horseradish peroxidase (MerkMillipore, Tumecula, CA) diluted 1:2,000 in PBST. After additional washes with PBST, the membrane was exposed to enhanced chemiluminescence reagent (MerckMillipore, Billerica, MA) and reactive signals visualized using autoradiography using Kodak BioMax film (Millipore Sigma, Burlington, MA)

### Immunohistochemistry

Paraffin-embedded sections of adult *O. viverrini* were de-paraffinized using xylene. Sections were rehydrated in an ethanol series, serial solution, with two incubations for each stage; 100%, 90%, 80% and 70% ethanol, 5 min each. Sections were immersed in citrate buffer (pH 6) and autoclaved for 10 min for antigen unmasking, followed by blocking with 3% H_2_O_2_ in methanol. Thereafter they were incubated overnight at 4°C in rabbit anti-*Ov*-PGRN-L627C641 peptide sera diluted 1:10, or pre-immunized rabbit IgG in PBS. Sections were probed with anti-rabbit IgG Fc monoclonal secondary antibody (HRP conjugate) (GenScript, Cat. No. A01856, Piscataway, NJ) diluted 1:1,000 in PBS. Peroxidase reaction products were visualized with 3,3’-diaminobenzidine (DAB) (Sigma-Aldrich, St Louis, MO). Counterstaining was performed with Mayer’s hematoxylin for 5 min. A positive signal was indicated by a reddish-brown color under light microscopy.

### Cellular proliferation

The human cholangiocyte cell line, H69 was maintained as described [29]. H69 cells were cultured in the presence of the *Ov*-PGRN-L627C641 specific peptide. Briefly, 1.5×10^3^ H69 cells were seeded into wells of a 24 well cell culture plate (SPL Life Sciences, Korea) and cultured with complete medium for 24 hours. Complete medium is defined as Dulbecco’s modified Eagle’s medium (DMEM)/Ham-F12 supplemented with 100 u/ml penicillin-streptomycin, 25 μg/ml adenine, 5 μg/ml insulin, 1 μg/ml epinephrine, 13.6 ng/ml T3-T, 10 ng/ml epidermal growth factor and 0.62 μg/ml hydrocortisone, sterile-filtered through a 0.22 mm membrane, and then 10% fetal bovine serum was added [30]. Cells were then fasted for 4–6 hours in low growth factor media (DMEM/Ham-F12 supplemented with 100 U/ml penicillin-streptomycin with one-twentieth of the growth factor contents of complete media). The cells were cultured with 0.8 and 1.6 mM PGRN peptide, or 0.2 mM recombinant *Ov*-GRN-1 [8, 9], or vehicle control PBS in low growth factor media for 24 and 48 hours. The viable cell number was determined using an tetrazolium salt (3-(4,5-dimethylthiazol-2-yl)-2,5-diphenyltetrazolium bromide (MTT) assay with absorbance 570 nm as per manufacturer’s instructions (Invitrogen, Oregon, USA). The three replicate experiments were assayed for each condition. Cell number was determined at 570 nm using a plate reader (Asys UVM340, Biochrom, Cambridge, UK). The concentrations were established using a standard curve before transforming into relative proliferation compared to control groups. Cell proliferation assays were carried out in triplicate. The data were presented as the mean ± SE of three independent replicates using GraphPad Prism software. One-way ANOVA with a post-hoc tukey test was used for statistical significance comparison, *p* ≤0.05.was considered to be statistically significant.

### IL-6 and IL-8

IL-6 and IL-8 gene expression levels from the H69 cholangiocyte cell line co-cultured with *Ov*-PGRN specific peptide was measured by qRT-PCR. Briefly, H69 cells were treated with *Ov*-PGRN specific peptide as described above and subsequently total RNA of harvested cells incubated with 0.8, 1.6 mM of *Ov*-PGRN specific peptide was extracted using TriZol^®^Reagent following company instructions (ThermoFisher Scientific, Burlington, MA). 500 ng of total RNA was converted to cDNA by using a RevertAid first strand cDNA synthesis kit (Thermo Fisher Scientific, Waltham, MA) for qPCR templates. cDNA was amplified using PCR with gene-specific primers designed to amplify a portion of the coding sequences. qPCR was performed with biological duplicate samples using a SYBR Green kit (Maxima SyBr green qPCR master mix, ThermoFisher Scientfic, Vilnius, EU) in a thermal cycler (Light Cycler 480 II, Roche Diagnostics GmbH, Mannheim, Germany). Detail of primer sequences for IL-6 (forward; 5’-ACCCCTGACCCAACCACAAAT-3’, reverse; 5’-CCTTAAAGCTGCGCAGAATGAGA-3’), IL-8 (forward; 5’-GTGCAGTTTTGCCAAGGAGT-3’, reverse; 5’-CTCTGCACCCAGTTTTCCTT-3’)[30]. The gene expressions were normalized with internal control using Beta-actin (forward; 5’-TCCCTGGAGAAGAGCTACGA, reverse; 5’AGCACTGTGTTGGCGTACAG)[27].PCR reactions consisted of 12.5 μl of SYBR Green Master Mix (ThermoFisher Scientific, Vilnius, EU), 0.5 μl (10 mM) each of forward and reverse primers, 1 μl (equivalent to 50 ng of total RNA) of first- stand cDNA and water to a final volume of 25 μl. PCR cycling conditions consisted of initiation with pre-heat for one cycle at 95°C for 10 min followed by 40 cycles of denaturation at 95°C for30 sec, annealing at 55°C for 30 sec, extension at 72°C for 45 sec, and a final extension at 72°C for 10 min. Data are presented as the mean ± standard error. Differences between groups were assessed using Student’s *t*-test (GraphPad Prism Software, www.graphpad.com); *p* ≤0.05.was considered statistically significant.

## Results

### Characteristics of O. viverrini progranulin

**Figure 1:**
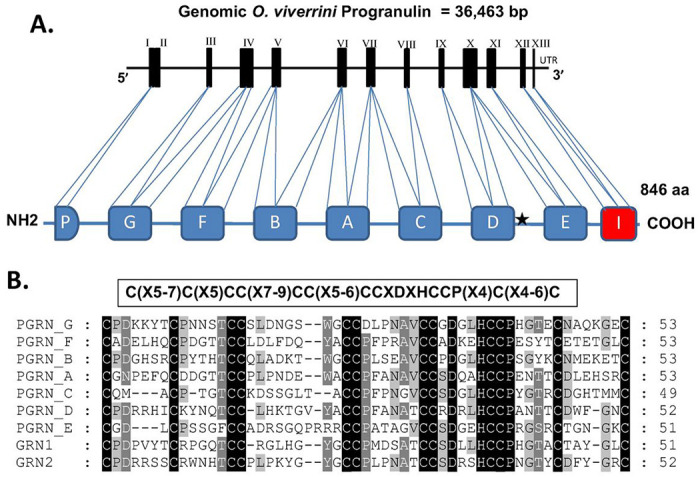
Secondary structure of *O. viverrini* progranulin gene and protein. (A) Structure of genomic sequence of *Ov*-PGRN consists of 13 exons and 12 introns and the schematic of *Ov*-PGRN protein composed of seven and a half repeats of the granulin/epithelin motif. One half granulin domain is indicated with a P, and the 7 complete granulin domains were designated A to G. Consensus sequences of 12-cysteine granulin/epithelin motif, C(X5-7)C(X5)CC(X7-9)CC(X5-6)CCXDXHCCP(X4)C(X4-6)C was identified in the complete domains A to G. An incomplete granulin/epithelin motif with 10-cysteine in exon 12-13 is indicated with I. Position of *Ov*-PGRN specific peptide (*Ov*-PGRN-L627C641) is indicated with black star between domain D and E. (B) Multiple sequence alignment of single granulin motif A to G compared with *Ov*-GRN-1 and *Ov*-GRN-2.

The genomic sequence of progranulin of *O. viverrini*(*Ov*-PGRN) was identified from the whole genome shotgun sequence database (Nucleotides 46,266-9,212 of GenBank Sequence ID: JACJ01021739.1) [10]. The genomic structure of *Ov*-PGRN was 36,463 nucleotides composed of 13 exons and 12 introns with an upstream promoter which is compatible with the expression of this gene (Fig. 1A). The *O. viverrini* progranulin (*Ov*-PGRN) mRNA sequence was identified from a partial mRNA sequence encoding a hypothetical protein (GenBank accession number XM_009176368) [10]. The mRNA sequence is 2,768 bp in length and encoded 846 amino acids with a signal peptide containing a predicted cleavage site between amino acids 38 and 39: VLS-GD (Supplemental Fig. 1). *Ov*-PGRN contains specific characteristics of one half and seven complete and highly conserved 12-cysteine granulin/epithelin motifs: C(X5-7)C(X5)CC(X7-9)CC(X5-6)CCXDXHCCP(X4)C(X4-6)C [11] (Fig. 1A and B). The individual granulin/epithelin motifs (A-G) show sequence similarities between 53–67%.

**Figure 2:**
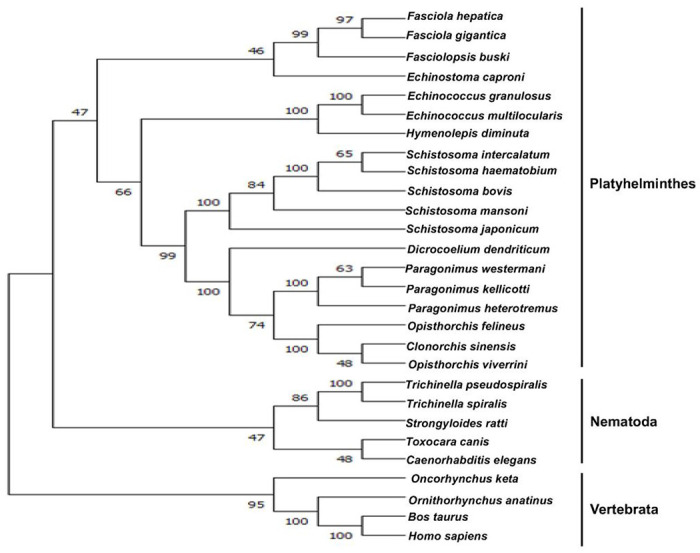
Maximum likelihood tree showing the relationship between progranulin of *O. viverrini* and homologou progranulin protein sequences from various helminths and vertebrates. Bootstrap values of 1,000 replicates are provided at the nodes of the branches.

Phylogenetic relationship of *Ov*-PGRN and other PGRNs from various species indicated that *Ov*-PGRN formed the closest relationship with PGRN of the closely related carcinogenic flukes *Clonorchis sinensis* and *O. felineus* (Fig. 2). PGRN formed major branches in which the Platyhelminthes, Nematoda, and Vertebrata all grouped within their clades.

### Progranulin is highly expressed in distinct developmental stages of O. viverrini

**Figure 3:**
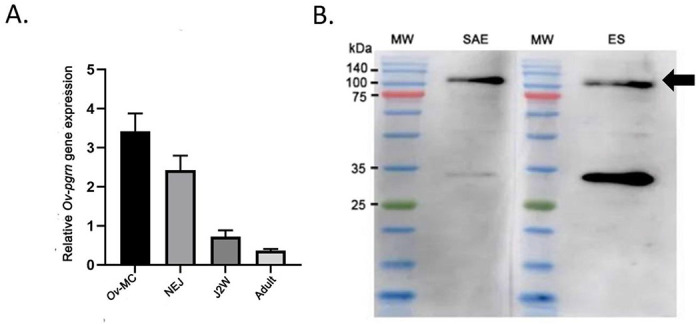
Detection of *Ov-pgrn* gene and *Ov*-PGRN protein in *O. viverrini*. (A) Relative expression by RT-qPCR of *Ov-pgrn*gene in adult, 2 weeks old fluke (J2W), newly excysted juvenile (NEJ) and metacercaria (*Ov*-MC). The evaluation of *Ov*-pgrn expression was performed by relative gene expression analysis using endogenous actin gene as a reference. (B) Detection of *O*. *viverrini* PGRN by western blot. Western blot shows somatic adult extract, SAE (2 ug) and ES (5 ug) from adult fluke on nitrocellulose membrane probed with anti-*Ov*-PGRN-L627C641 peptide followed by HRP-conjugated anti-rabbit IgG secondary antibody. A specific band was detected for progranulin/PGRN at approximately 92 kDa (as indicated by arrow). Molecular weight marker (MW) is indicated.

The developmental expression profile of *Ov-pgrn* in *O. viverrini* was evaluated using quantitative RT-PCR, and protein expression profile was assessed using immunohistochemistry. *Ov-pgrn* mRNA was expressed in metacercaria, newly excysted juvenile (NEJ), two-week old juvenile (J2W) and adult fluke (Fig. 3). The highest *Ov-pgrn* gene expression level was shown in metacercaria and the lowest levels were in NEJ, J2W and adult, respectively (Fig. 3A). Western blot analysis detected a band of 100–110 kDa, slightly larger than the expected size of *Ov*-PGRN (92 kDa) in both worm lysate and ES products (Fig. 3B). The greater observed size is likely due to the 10 putative N-linked glycosylation sites identified in the predicted protein (Supplemental Fig. 1).

**Figure 4:**
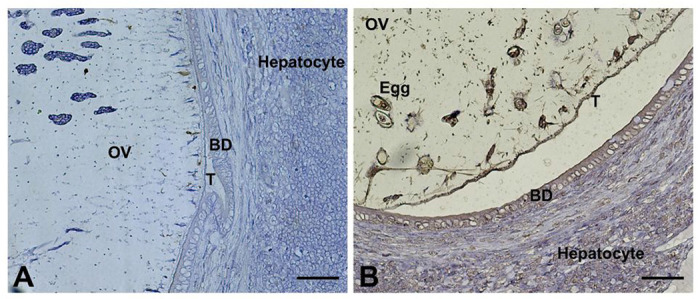
Immunohistochemical localization of *Ov*-PGRN in adult *O. viverrini*. Thin histological sections of adult *O. viverrini* in the bile ducts of infected hamsters probed with (A) with control IgG and (B) probed with rabbit anti-*Ov*-PGRN-L627C641 peptide. OV = worm, BD = bile duct epithelium, T= tegument. Scale bar = 50 mm.

Immunohistochemical localization of the liver of infected hamster probed with rabbit anti-*Ov*-PGRN-L627C641 peptide revealed expression of *Ov*-PGRN on the tegument, parenchymal and eggs in the uterus of the adult fluke in hamster bile ducts (Fig. 4). The signal also was detected in bile duct cells of the infected hamster (Fig. 4).

### Effect of Ov-PGRN peptide on human bile duct cell proliferation

**Figure 5:**
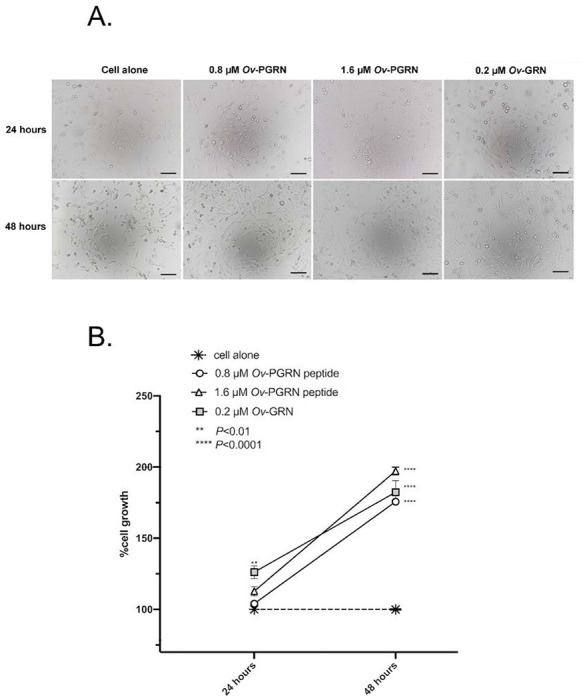
*Opisthorchis viverrini* progranulin peptide stimulates the proliferation of human cholangiocytes *in vitro*. H69 cells were incubated with 0.8 and 1.6 mM of *Ov*-PGRN synthetic peptide and observed cell relative proliferation at 24 and 48 h. Recombinant Ov-GRN-1 expressed from *E. coli* and refolded to an active form at 0.2 mM to stimulate cell proliferation is a positive control. (A) H69 cholangiocytes cultured in the presence or absence of progranulin peptide. Scale bar is 50 μm. (B) Percent cell proliferation from different concentrations of peptide on human H69 cholangiocytes using the MTT assay. Error bars reveal the standard error of triplicate biological replicates. The symbols ** and **** denote statistical significance at the level of *p* ≤ 0.01 and *p* ≤ 0.0001, respectively.

H69 cells were incubated with 0.8 and 1.6 μM of *Ov*-PGRN-L627C641 peptide and cell proliferation was measured with the MTT assay at 24 and 48 h. H69 cells incubated with 0.2 μM of *Ov*-GRN-1 and culture media were used as a control groups. The results showed that H69 cells incubated with 0.8 and 1.6 μM of *Ov*-PGRN-L627C641 peptide underwent significantly increased cell proliferation only at 48 hrs (160 and 180%, respectively) when compared with culture media alone control (Fig. 5B). H69 cells incubated with 0.2 μM of *Ov*-GRN-1 recombinant protein also underwent significantly increased cell proliferation when compared with culture media alone, as described previously [9].

### Pulsing cholangiocytes with Ov-PGRN peptide-stimulated expression of IL6 and IL8.

**Figure 6:**
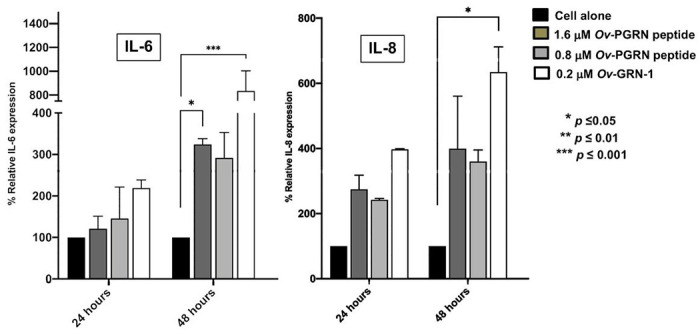
Quantitative RT-PCR detecting relative gene expression levels of IL6 and IL8 in human cholangiocytes, H69 incubated with *Ov*-PGRN-L627C641 peptide. The *O. viverrini* actin gene was used as an internal control. *, **, *** Denoted statistical significance at the level of *p* ≤ 0.05, *p* ≤ 0.01 and ≤ 0.001, respectively.

To evaluate the inflammatory cytokine production that occurs in response to *O. viverrini* progranulin stimulation. The response of cultured human bile duct cell lines to *Ov*-PGRN-L627C641 peptide stimulation was investigated. Quantitative RT-PCR was applied to evaluate the mRNA expression level of cytokine from the cells. H69 cells were incubated with 0.8 and 1.6 μM of *Ov*-PGRN-L627C641 peptide. The relative expression was compared with actin house-keeping gene. The mRNA expression levels of IL-6 and IL-8 were slightly increased in H69 cells incubated with either 0.8 or 1.6 μM of *Ov*-PGRN -L627C641 peptide at 48 h incubation compared to controls without peptide (Fig. 6).

## Discussion

It has been well established that *Ov*-GRN-1, liver fluke granulin, stimulates proliferation of cholangiocytes lining the biliary tract [7, 8, 31]. The genome of *O*. *viverrini*contains two genes encoding single granulin domains termed *Ov-grn-1* and *Ov-grn-2 and* also the multiple granulin domain gene, *Ov-pgrn* [10]. The *Ov*-PGRN glycoprotein exhibits homology to human progranulin which has seven and a half conserved granulin domains [11, 20]. Here, we described the intact progranulin from the genome of *O*. *viverrini*, which we predict consists of one half and seven complete tandem repeats of a 12-cysteine module granulin domain and one incomplete granulin domain. In addition, we describe the predicted structure and likely roles of the liver fluke progranulin.

The *O. viverrini* progranulin showed typical characteristics of the repeat granulin motif. Progranulins from other species stimulate cellular proliferation when intact or when cleaved into single granulin units [20, 32]. Detection of progranulin transcripts in larval and adult stages of *O. viverrini* revealed that intact progranulin is active. Adult liver flukes graze on the biliary epithelium where they secrete ES products and stimulate a biliary mucosal immune response. Cell-mediated immune responses were investigated and showed that the animal infected with *O. viverrini* has granulomatous inflammation of bile duct by modified macrophage [22]. IL-6 and IL-8 were increased in hamster and human with hepatobiliary abnormalities [33–35].Treatment with progranulin showed decreasing of liver fibrosis and inflammation in mice and macrophage [36]. While intact human PGRN showed evidence of suppresses inflammation by blocking TNF-α receptors and signaling [13]. Proteolytic enzymes degrade PGRN to granulin domain peptides which may enhance inflammation by stimulating the secretion of the chemokine interleukin-8 [13]. Our findings revealed that *O. viverrini* PGRN peptide increase expression of IL-6 and IL-8 that may involve inflammation of bile duct. We note that access to the genomic structure included the sequences of the introns and exons of *O. viverrini* progranulin gene will facilitate for programmed gene editing and functional genomics analysis, in like fashion to our studies of *Ov-grn-1* [7].

The excretory and secretory molecules of the parasite directly promoting cell proliferation, both innate and adaptive host inflammatory responses in chronic infection contribute to infection-induced malignancy (Sripa et al., 2007). Molecules in ES products stimulate naive T-cell with Toll-like receptor 4 signaling and express IL6 and IL8 [30]. Upregulation of the proinflammatory transmembrane molecule, TLR4, has been reported in cholangiocytes (H69 cells) cocultured with ES products of *O. viverrini* [30]. TLR4 overexpression has also been observed in the biliary epithelium of *O. viverrini* infected humans in situ. The parasite products also induce IkB-a degradation in a MyD88-dependent manner and activate nuclear factor kappa B nuclear translocation, leading to the increased expression and secretion of the strong chemoattractant chemokine IL-8 and proinflammatory cytokine IL-6 [30]. TLR4 is a transmembrane protein, member of the toll-like receptor family, which belongs to the pattern recognition receptor (PRR) family. Its activation leads to an intracellular signaling pathway NF-κB and inflammatory cytokine production which is responsible for activating the innate immune system. These results demonstrated that *O. viverrini* PGRN stimulated human cholangiocytes and initiate innate mucosal immunity/inflammatory via TLR4 pathway. Given that progranulin of the *O. viverrini* was identified in ES product and seems involved in proliferation and inflammation, future research should explore if *Ov*-PGRN is involved in carcinogenesis similar to the single granulin protein, *Ov*-GRN-1 [6–8].

## Data Availability

All data generated or analysed during this study are included in this published article [and its supplementary information files].

## References

[1] IARC (2012) Biological agents. Volume 100 B. A review of human carcinogens. IARC monographs on the evaluation of carcinogenic risks to humans 100(Pt B):1–441. PMC4781184 23189750

[2] Brindley PJ, Loukas A (2017) Helminth infection-induced malignancy. PLoS pathogens 13(7):e1006393. 10.1371/journal.ppat.1006393 28750101 PMC5531427

[3] Brindley PJ, Bachini M, Ilyas SI, Khan SA, Loukas A, Sirica AE, (2021) Cholangiocarcinoma. Nature reviews Disease primers 7(1):65. 10.1038/s41572-021-00300-2 PMC9246479 34504109

[4] Haugen B, Karinshak SE, Mann VH, Popratiloff A, Loukas A, Brindley PJ, (2018) Granulin Secreted by the Food-Borne Liver Fluke *Opisthorchis viverrini* Promotes Angiogenesis in Human Endothelial Cells. Frontiers in medicine 5:30. 10.3389/fmed.2018.00030 29503819 PMC5820972

[5] Chaiyadet S, Sotillo J, Krueajampa W, Thongsen S, Smout M, Brindley PJ, (2022) Silencing of *Opisthorchis* *viverrini* Tetraspanin Gene Expression Results in Reduced Secretion of Extracellular Vesicles. Frontiers in cellular and infection microbiology 12:827521. 10.3389/fcimb.2022.827521 35223551 PMC8875506

[6] Chaiyadet S, Tangkawattana S, Smout MJ, Ittiprasert W, Mann VH, Deenonpoe R, (2022) Knockout of liver fluke granulin, Ov-grn-1, impedes malignant transformation during chronic infection with *Opisthorchis viverrini*. PLoS pathogens 18(9):e1010839. 10.1371/journal.ppat.1010839 36137145 PMC9531791

[7] Arunsan P, Ittiprasert W, Smout MJ, Cochran CJ, Mann VH, Chaiyadet S, (2019) Programmed knockout mutation of liver fluke granulin attenuates virulence of infection-induced hepatobiliary morbidity. eLife 8. 10.7554/eLife.41463 PMC6355195 30644359

[8] Smout MJ, Laha T, Mulvenna J, Sripa B, Suttiprapa S, Jones A, (2009) A granulin-like growth factor secreted by the carcinogenic liver fluke, *Opisthorchis viverrini*, promotes proliferation of host cells. PLoS pathogens 5(10):e1000611. 10.1371/journal.ppat.1000611 19816559 PMC2749447

[9] Smout MJ, Sotillo J, Laha T, Papatpremsiri A, Rinaldi G, Pimenta RN, (2015) Carcinogenic Parasite Secretes Growth Factor That Accelerates Wound Healing and Potentially Promotes Neoplasia. PLoS pathogens 11 (10):e1005209. 10.1371/journal.ppat.1005209 26485648 PMC4618121

[10] Young ND, Nagarajan N, Lin SJ, Korhonen PK, Jex AR, Hall RS, (2014) The *Opisthorchis viverrini* genome provides insights into life in the bile duct. Nature communications 5:4378. 10.1038/ncomms5378 PMC4104445 25007141

[11] Palfree RG, Bennett HP Bateman A (2015) The Evolution of the Secreted Regulatory Protein Progranulin. PloS one 10(8):e0133749. 10.1371/journal.pone.0133749 26248158 PMC4527844

[12] Bhandari V, Bateman A (1992) Structure and chromosomal location of the human granulin gene. Biochemical and biophysical research communications 188(1):57–63. 10.1016/0006-291x(92)92349-3 1417868

[13] Toh H, Chitramuthu BP Bennett HP Bateman A (2011) Structure, function, and mechanism of progranulin; the brain and beyond. Journal of molecular neuroscience : MN 45(3):538–548. 10.1007/si2031-011-9569-4 21691802

[14] He Z, Bateman A (2003) Progranulin (granulin-epithelin precursor, PC-cell-derived growth factor, acrogranin) mediates tissue repair and tumorigenesis. J Mol Med (Berl) 81 (10):600–612. 10.1007/S00109-003-0474-3 12928786

[15] Zhu J, Nathan C, Jin W, Sim D, Ashcroft GS, Wahl SM, (2002) Conversion of proepithelin to epithelins: roles of SLPI and elastase in host defense and wound repair. Cell 111 (6):867–878. 10.1016/s0092-8674(02)01141-8 12526812

[16] Bansal PS, Smout MJ, Wilson D, Cobos Caceres C, Dastpeyman M, Sotillo J, (2017) Development of a Potent Wound Healing Agent Based on the Liver Fluke Granulin Structural Fold. Journal of medicinal chemistry 60(10):4258–4266. 10.1021/acs.jmedchem.7b00047 28425707 PMC12212975

[17] Dastpeyman M, Bansal PS, Wilson D, Sotillo J, Brindley PJ, Loukas A, (2018) Structural Variants of a Liver Fluke Derived Granulin Peptide Potently Stimulate Wound Healing. Journal of medicinal chemistry 61 (19):8746–8753. 10.1021/acs.jmedchem.8b00898 30183294 PMC12212974

[18] Wang C, He Q, Yin Y, Wu Y, Li X (2021) *Clonorchis sinensis* Granulin Promotes Malignant Transformation of Hepatocyte Through EGFR-Mediated RAS/MAPK/ERK and PI3K/Akt Signaling Pathways. Frontiers in cellular and infection microbiology 11:734750. 10.3389/fcimb.2021.734750 34858869 PMC8631275

[19] Petkau TL, Leavitt BR (2014) Progranulin in neurodegenerative disease. Trends in neurosciences 37(7):388–398. 10.1016/j.tins.2014.04.003 24800652

[20] Bateman A, Bennett HP (2009) The granulin gene family: from cancer to dementia. BioEssays : news and reviews in molecular, cellular and developmental biology 31(11):1245–1254. 10.1002/bies.200900086 19795409

[21] Sripa B, Kaewkes S (2002) Gall bladder and extrahepatic bile duct changes in Opisthorchis viverrini-infected hamsters. Acta tropica 83(1):29–36. 10.1016/s0001-706x(02)00052-9 12062790

[22] Sripa B, Kaewkes S (2000) Localisation of parasite antigens and inflammatory responses in experimental opisthorchiasis. International journal for parasitology 30(6):735–740. 10.1016/S0020-7519(00)00054-0 10856508

[23] Altschul SF, Gish W, Miller W, Myers EW, Lipman DJ (1990) Basic local alignment search tool. Journal of molecular biology 215(3):403–410. 10.1016/S0022-2836(05)80360-2 2231712

[24] Hall T (1999) BioEdit: a user friendly biological sequence alignment editor and analysis program for Windows 95/98/NT. Nucleic Acids Symposium Series 41:95–98.

[25] Thompson JD, Higgins DG, Gibson TJ (1994) CLUSTAL W: improving the sensitivity of progressive multiple sequence alignment through sequence weighting, position-specific gap penalties and weight matrix choice. Nucleic Acids Res 22(22):4673–4680. 7984417 10.1093/nar/22.22.4673 PMC308517

[26] Tamura K, Stecher G, Kumar S (2021) MEGA11: Molecular Evolutionary Genetics Analysis Version 11. Molecular biology and evolution 38(7):3022–3027. 10.1093/molbev/msab120 33892491 PMC8233496

[27] Papatpremsiri A, Smout MJ, Loukas A, Brindley PJ, Sripa B, Laha T (2015) Suppression of Ov-grn-1 encoding granulin of *Opisthorchis viverrini* inhibits proliferation of biliary epithelial cells. Experimental parasitology 148:17–23. 10.1016/j.exppara.2014.11.004 25450776 PMC4277937

[28] Schmittgen TD, Livak KJ (2008) Analyzing real-time PCR data by the comparative C(T) method. Nature protocols 3(6):1101–1108. 10.1038/nprot.2008.73 18546601

[29] Arunsan R, Chaidee A, Cochran CJ, Mann VH, Tanno T, Kumkhaek C, (2020) Liver fluke granulin promotes extracellular vesicle-mediated crosstalk and cellular microenvironment conducive to cholangiocarcinoma. Neoplasia 22(5):203–216. 10.1016/j.neo.2020.02.004 32244128 PMC7118280

[30] Ninlawan K, O’Hara SP, Splinter PL, Yongvanit P, Kaewkes S, Surapaitoon A, (2010) *Opisthorchis viverrini* excretory/secretory products induce toll-like receptor 4 upregulation and production of interleukin 6 and 8 in cholangiocyte. Parasitology international 59(4):616–621. 10.1016/j.parint.2010.09.008 20887801 PMC3319364

[31] Wang C, Lei H, Tian Y, Shang M, Wu Y, Li Y, (2017) *Clonorchis sinensis* granulin: identification, immunolocalization, and function in promoting the metastasis of cholangiocarcinoma and hepatocellular carcinoma. Parasites & vectors 10(1):262. 10.1186/s13071-017-2179-4 28545547 PMC5445496

[32] Abella V, Pino J, Scotece M, Conde J, Lago R, Gonzalez-Gay MA, (2017) Progranulin as a biomarker and potential therapeutic agent. Drug discovery today 22(10):1557–1564. 10.1016/j.drudis.2017.06.006 28651064

[33] Sripa B, Mairiang E, Thinkhamrop B, Laha T, Kaewkes S, Sithithaworn P (2009) Advanced periductal fibrosis from infection with the carcinogenic human liver fluke *Opisthorchis viverrini* correlates with elevated levels of interleukin-6. Hepatology 50(4):1273–1281. 10.1002/hep.23134 19676135 PMC3682769

[34] Sripa B, Thinkhamrop B, Mairiang E, Laha T, Kaewkes S, Sithithaworn R (2012) Elevated plasma IL-6 associates with increased risk of advanced fibrosis and cholangiocarcinoma in individuals infected by *Opisthorchis viverrini*. PLoS neglected tropical diseases 6(5):e1654. 10.1371/journal.pntd.0001654 22629477 PMC3358341

[35] Sripa B, Jumnainsong A, Tangkawattana S, Haswell MR (2018) Immune Response to *Opisthorchis viverrini* Infection and Its Role in Pathology. Advances in parasitology 102:73–95. 10.1016/bs.apar.2018.08.003 30442311

[36] Yoo W, Lee J, Noh KH, Lee S, Jung D, Kabir MH, (2019) Progranulin attenuates liver fibrosis by downregulating the inflammatory response. Cell death & disease 10(10):758. 10.1038/S41419-019-1994-2 31591383 PMC6779917

